# Single-Band-Notched Ultra-Wideband Low-Sidelobe Planar Array Antenna for Millimeter-Wave Applications

**DOI:** 10.3390/mi17050624

**Published:** 2026-05-19

**Authors:** Yuanjun Shen, Tianling Zhang

**Affiliations:** National Key Laboratory of Radar Detection and Sensing, Xidian University, Xi’an 710071, China; yuanjun.shen@xidian.edu.cn

**Keywords:** ultra-wideband array antenna, band-notched antenna, low sidelobe level, millimeter-wave antenna, planar array, Taylor feeding network

## Abstract

A single-band-notched ultra-wideband (UWB) low-sidelobe planar array antenna for millimeter-wave (mmWave) applications is presented. The antenna element employs a planar dipole excited through an H-shaped coupling slot to achieve broadband impedance matching, while a centrally loaded parasitic patch acts as a half-wavelength resonator to generate a controllable notch band. Additional parasitic patches are introduced to recover the high-frequency matching without degrading the notch response. An 8×8 array is then developed using a Taylor-weighted feed network implemented with three classes of 1-to-4 microstrip power dividers. Measured results show that the array operates from 19.0 to 45.0 GHz with VSWR<2, while providing a rejection band from 35.0 to 38.5 GHz. The notch suppresses the realized gain by about 5 dB around 37.0 GHz, the peak gain reaches 20.5 dBi in the passband, and average sidelobe levels better than −17 dB are obtained. The proposed design provides a practical approach for combining ultra-wide bandwidth, in-band interference rejection, and low-sidelobe radiation in a compact mmWave planar array.

## 1. Introduction

Ultra-wideband (UWB) planar array antennas operating at millimeter-wave (mmWave) frequencies have attracted considerable attention over the past decade, driven by the rapid development of fifth-generation (5G) and emerging sixth-generation (6G) wireless communication systems [1,2]. The abundant spectrum resources available in the mmWave bands offer the potential for multi-gigabit-per-second data rates, enabling a wide range of applications including high-resolution radar, satellite communications, vehicular networking, and high-throughput wireless links [2,3]. To fully exploit these spectral resources, antenna systems are required to provide broad operating bandwidths, high gain, and well-controlled radiation patterns while maintaining compact form factors compatible with modern platforms.

However, ultra-wideband operation also increases the possibility of coupling unwanted signals from adjacent or coexisting mmWave services. This issue is relevant to the 37 GHz region, where the 37.0–40.0 GHz range includes the 5G NR n260 band [4], and the 37.0–38.6 GHz range is associated with fixed/mobile services and shared or flexible-use spectrum frameworks [5]. Therefore, for broadband mmWave front ends, an antenna-level rejection band around 37 GHz can provide useful pre-suppression of potential sub-band interference while maintaining radiation performance in the adjacent passbands.

Related studies on programmable and time-modulated metasurfaces have also shown that radar and sensing environments can be manipulated in increasingly flexible ways, for example through range–Doppler modulation and SAR imaging response control [6,7,8,9]. Although these studies address different hardware concepts from the antenna array considered here, they highlight the growing complexity of electromagnetic environments and further motivate broadband antenna front ends with selective spectral suppression.

Over the years, a variety of UWB antenna element topologies have been investigated for mmWave applications. The Vivaldi antenna, first introduced by Gibson [10], has been widely employed because of its inherently broadband endfire radiation characteristics and ease of fabrication [11,12]. However, Vivaldi elements tend to exhibit relatively large physical profiles and are less amenable to planar two-dimensional array configurations. To address these limitations, planar radiating elements have been extensively studied, including magnetoelectric dipoles [13,14], tightly coupled dipole arrays [15,16], slot-loaded circular patches [17], folded dipoles [18,19], and stub-loaded dipoles fed by substrate-integrated coaxial lines (SICLs) [20,21]. Among these, planar dipole-based designs are particularly attractive for array integration because they offer a favorable tradeoff between bandwidth, cross-polarization performance, and structural simplicity. Wideband circularly polarized elements, such as the Bold-C spiral structure and C-shaped strip antennas, have also demonstrated promising performance for mmWave array applications requiring polarization diversity [22,23].

In many practical scenarios, such as radar surveillance, satellite communication, and electronic warfare, low sidelobe levels (SLLs) are highly desirable to minimize interference and improve signal-to-noise ratios. Classical amplitude tapering techniques, including the Dolph–Chebyshev and Taylor distributions, remain the standard tools for sidelobe control [24,25]. At mmWave frequencies, the implementation of low-sidelobe feed networks presents additional challenges due to the compact element spacing and the sensitivity of transmission-line discontinuities. A number of recent studies have therefore explored broadband low-sidelobe arrays based on slow-wave feed networks, unequal power dividers, SIW technologies, ridge gap waveguides, and Rotman-lens architectures [26,27,28,29,30,31,32,33,34,35].

While UWB antennas offer wide spectral coverage, the broad operating bandwidth inevitably encompasses frequency bands allocated to other narrowband wireless services. In the sub-6 GHz regime, this coexistence problem has motivated a rich body of work on band-notched UWB antennas. Common notch-generation techniques include etching resonant slots, loading parasitic elements, embedding open-loop or split-ring resonators, and introducing coupled-resonator structures for sharper selectivity [36,37,38,39,40,41]. In the mmWave regime, however, the design of band-notched antennas—especially within array configurations—has received comparatively less attention. Recent studies have reported directly connected linear arrays and conformal UWB antennas with notch characteristics [42,43], while fragment-type etched patterns have been used to sharpen the notch response [44]. Nonetheless, achieving a well-defined band-notch function within a planar UWB array antenna while maintaining low sidelobe performance remains insufficiently addressed. Most existing band-notched UWB antennas are implemented as single radiators or low-frequency UWB antennas, where the array-level feeding network, aperture taper, and finite-aperture effects are not the primary design constraints. Conversely, many reported mmWave low-sidelobe arrays focus on broadband radiation and amplitude-tapered feeding networks, but they usually do not incorporate an intentional in-band rejection function.

The simultaneous realization of ultra-wide bandwidth, band-notch functionality, and low sidelobe levels in a planar mmWave array is therefore a multifaceted design challenge. At the element level, the radiator must provide broadband impedance matching together with effective frequency rejection within a prescribed stopband. At the array level, the feeding network must deliver accurately controlled amplitude distributions for sidelobe suppression while accommodating the spectral gap introduced by the notch.

**Figure 1:**
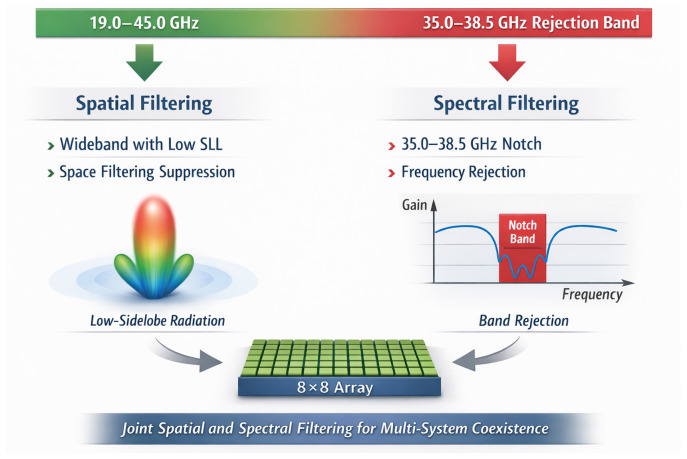
Conceptual illustration of the proposed array’s joint spatial and spectral filtering capabilities.

This paper addresses the above challenge by presenting a single-band-notched UWB low-sidelobe planar array antenna for mmWave applications. As illustrated conceptually in Figure 1, the proposed design integrates spectral filtering and spatial filtering within a compact planar mmWave aperture. The antenna element employs a slot-coupled planar dipole for ultra-wideband impedance matching and a centrally loaded parasitic patch as a half-wavelength resonator for controllable notch generation, while the 8×8 array uses a Taylor-weighted corporate feeding network for low-sidelobe radiation. Compared with conventional band-notched UWB antennas that mainly focus on element-level spectral rejection, this work further considers the interaction among the notch element, finite array aperture, and amplitude-tapered feeding network. The fabricated prototype demonstrates an operating bandwidth from 19.0 to 45.0 GHz, a 35.0–38.5 GHz rejection band, and average sidelobe levels better than −17 dB across 19.0–35.0 GHz.

## 2. Antenna Design

**Figure 2:**
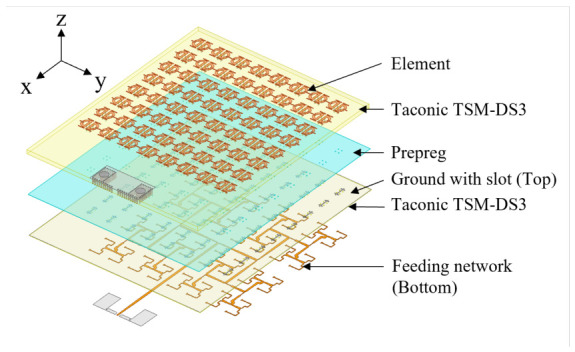
Geometry of the proposed 8×8 single-band-notched array antenna.

The topology of the proposed 8×8 single-band-notched UWB low-sidelobe planar array antenna is shown in Figure 2. The array comprises two stacked dielectric layers of Taconic TSM-DS3 with a relative permittivity of $\varepsilon_{r}=3$ and a loss tangent of tanδ=0.0011. The substrate thicknesses are 1.016 mm for substrate 1 and 0.127 mm for substrate 2, and the two layers are laminated using Prepreg FR27-0040-43F bonding material. The radiating elements are printed on the upper surface of substrate 1, while the ground plane with etched H-shaped coupling slots is located on the upper surface of substrate 2. The microstrip feeding network is printed on the lower surface of substrate 2. Two pairs of metallized through-vias penetrating all dielectric layers connect the radiating patches to the ground plane and facilitate fabrication. The overall array dimensions are $45\times 55\times 1.3\;{\mathrm{mm}}^{3}$.

Although the proposed array has a compact footprint and includes fine microstrip-line, coupling slot, and via features, all critical dimensions were defined according to the design-for-manufacturing rules provided by a professional RF/microwave PCB manufacturer experienced in high-frequency multilayer boards. Before fabrication, the minimum line width, line spacing, via diameter, annular ring, copper thickness, substrate thickness, and bonding-layer thickness were checked against the manufacturer’s process limits, and the Gerber files and stack-up were further reviewed by the manufacturer. Features close to the fabrication limits were adjusted when necessary. Therefore, the proposed geometry is a manufacturable mmWave multilayer PCB design rather than an idealized structure beyond practical fabrication capability.

From a system-level perspective, the array architecture integrates three functional blocks: (i) a broadband planar dipole element that provides ultra-wideband impedance matching through slot coupling, (ii) a parasitic-patch resonator that introduces a controllable notch band via half-wavelength resonance, and (iii) a Taylor-weighted feeding network that enforces the desired amplitude taper for low-sidelobe radiation. The following subsections detail the design of each block.

### 2.1. Band-Notched UWB Antenna Element

**Figure 3:**
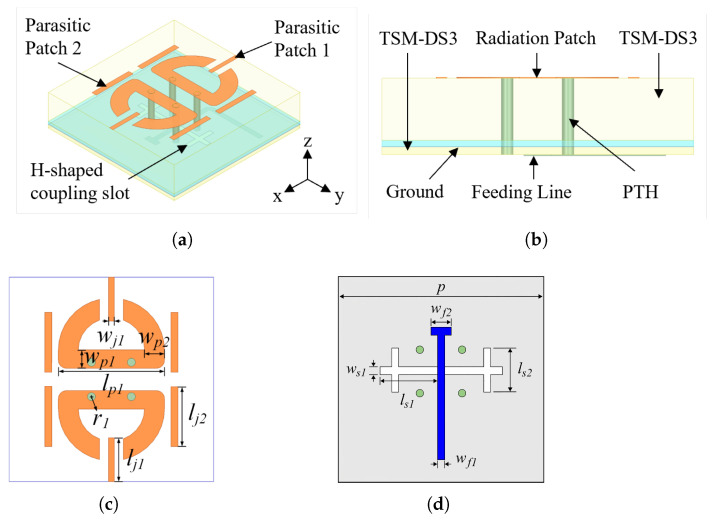
Geometry of the proposed antenna element: (**a**) perspective view, (**b**) side view, (**c**) top view, and (**d**) feeding structure.

The geometry of the proposed band-notched antenna element is shown in Figure 3, and its key dimensions are listed in Table 1. The radiating patch adopts a planar dipole structure and is printed on the top surface of substrate 1. An H-shaped coupling slot is etched on the ground plane, and a microstrip feed line on the lower surface of substrate 2 excites the radiating patch through the coupling slot. Between the tips of the two dipole arms, a parasitic patch (parasitic patch 1) is introduced to realize the band-notch function. On both sides of the radiating patch, two pairs of rectangular parasitic patches (parasitic patch 2) are loaded to improve the impedance matching at higher frequencies.

The H-shaped coupling slot and the centrally loaded parasitic patch are introduced based on an approximate mechanism-oriented analysis. The H-shaped slot can be interpreted using the aperture-coupled feeding mechanism. It transfers energy from the lower microstrip feed line to the upper planar dipole through electromagnetic coupling and introduces additional capacitive susceptance, which helps compensate for the inductive behavior of the dipole and feeding transition. Therefore, the coupling strength and the lower-band resonance can be adjusted by the slot dimensions without directly modifying the main dipole radiator.

The centrally loaded parasitic patch is placed in the gap between the two dipole arms, where strong local electric-field coupling exists. Through capacitive gap coupling, this patch can be effectively excited and behaves as a half-wavelength resonator around the target rejection frequency. At resonance, strong localized current is formed on the parasitic patch, and part of the electromagnetic energy is trapped by this resonant branch rather than being radiated by the main dipole. This leads to input-impedance deterioration and realized-gain reduction near the resonant frequency, thereby producing the desired band-notch response. Owing to the multilayer aperture-coupled configuration and the distributed radiation behavior of the element, this analysis is used as a resonator-based physical interpretation rather than a closed-form solution of the entire antenna structure.

#### 2.1.1. Step-by-Step Design Procedure

**Figure 4:**
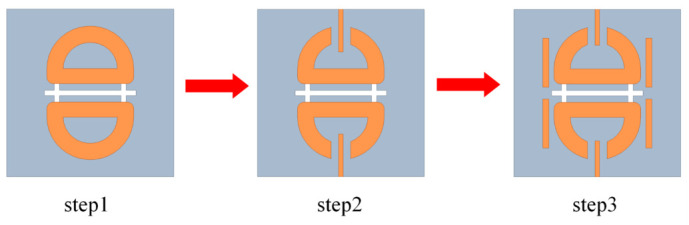
Step-by-step design evolution of the proposed band-notched UWB antenna element.

**Figure 5:**
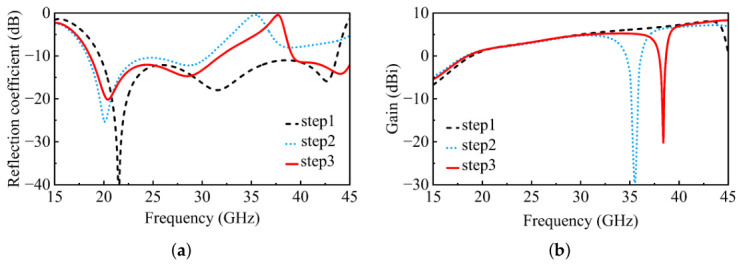
Simulated (**a**) reflection coefficients and (**b**) realized gains of the antenna element at each design step.

The design procedure is illustrated in Figure 4, and the corresponding simulated reflection coefficients and realized gains at each step are presented in Figure 5. All simulations are performed under periodic boundary conditions using Ansys HFSS.

In Step 1, a basic UWB planar dipole antenna element is designed, which achieves $|S_{11}|\;<-10$ dB from 19.58 to 43.71 GHz, corresponding to a relative bandwidth of 76.25%. As discussed above, the H-shaped coupling slot provides aperture coupling and capacitive compensation for the slot-coupled dipole. By optimizing the slot dimensions, the lower-frequency resonance is adjusted and the low-frequency matching is improved without significantly disturbing the high-frequency response.

In Step 2, parasitic patch 1 is introduced between the two dipole arms to realize the band-notch characteristic. Following the resonator-based interpretation discussed above, the patch is excited through capacitive gap coupling and forms a localized half-wavelength resonant current path near the target notch frequency. This resonant branch traps part of the electromagnetic energy and suppresses the radiation current on the main dipole, leading to input-impedance deterioration and realized-gain reduction near the notch frequency.

The total length *L* of the half-wavelength resonator and the center frequency $f_{0}$ of the rejection band satisfy

(1) $$L\approx \frac{\lambda_{g}}{2}=\frac{c}{2f_{0}\sqrt{\varepsilon_{e}}},$$

where *c* is the speed of light in free space, $\lambda_{g}$ is the guided wavelength, and $\varepsilon_{e}$ is the effective relative dielectric constant. Since the parasitic patch can be approximated as a microstrip resonator, $\varepsilon_{e}$ is estimated using the conventional microstrip effective-permittivity expression [45,46]

(2) $$\varepsilon_{e}=\frac{\varepsilon_{r}+1}{2}+\frac{\varepsilon_{r}-1}{2}F\left(W/H\right),$$

where

(3) $$F\left(u\right)=\left\{\begin{array}{cc} {\left(1+12/u\right)}^{-1/2}+0.04{\left(1-u\right)}^{2}, & u\leq 1,\\ {\left(1+12/u\right)}^{-1/2}, & u\geq 1, \end{array}\right.$$

where u=W/H, *H* is the substrate thickness, and *W* is the effective width of the resonant strip. Using $\varepsilon_{r}=3$ and the initial resonator geometry, the estimated value is $\varepsilon_{e}\approx 2.35$. Therefore, for $f_{0}=37.5$ GHz, the initial resonator length is calculated as L≈2.61 mm. This analytical value is used as the starting point for full-wave optimization, and the final optimized length slightly differs due to fringing fields and capacitive/inductive coupling around the parasitic patch.

It should be noted that $l_{j1}$ denotes the length of one parasitic-patch segment within a single unit cell. Under periodic continuation, the effective resonant current path is approximately formed by two adjacent segments; therefore, the effective resonator length can be estimated as $L_{\mathrm{eff}}\approx 2l_{j1}$, excluding fringing and coupling effects.

**Figure 6:**
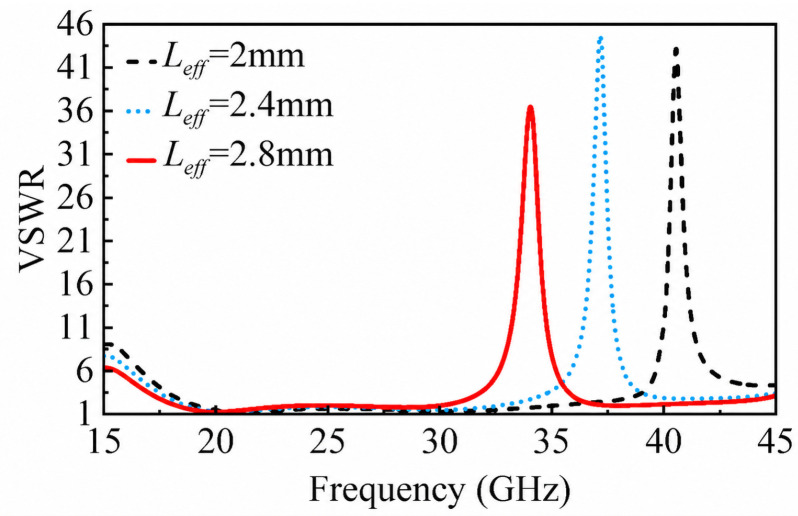
Effect of the effective resonator length $L_{\mathrm{eff}}$ on the VSWR, demonstrating the tunability of the notch center frequency.

Figure 6 shows the effect of the effective resonator length on the voltage standing-wave ratio (VSWR). As $l_{j1}$ increases from 1.0 to 1.4 mm, the corresponding effective resonator length $L_{\mathrm{eff}}$ increases from approximately 2.0 to 2.8 mm, and the notch center frequency shifts from 40.5 to 34.1 GHz. This monotonic downward shift is consistent with Equation (1). The slight deviation between the actual and theoretical lengths is attributed to the capacitive and inductive coupling effects between the patch and the ground plane.

**Figure 7:**
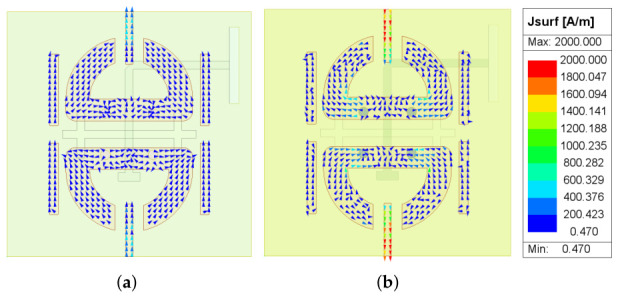
Surface-current distribution of the element at (**a**) 30 GHz (passband) and (**b**) 38 GHz (notch band).

The above notch mechanism is further confirmed by the surface-current distributions shown in Figure 7. At 30 GHz, which lies within the passband, the current flows predominantly along the dipole arms, enabling effective broadside radiation. In contrast, at 38 GHz, which lies inside the notch band, strong current is concentrated on parasitic patch 1. This confirms that parasitic patch 1 is resonantly excited and traps a significant portion of the electromagnetic energy, thereby suppressing the radiation current on the main dipole and producing the desired band rejection.

Following Step 2, the introduction of parasitic patch 1 deteriorates the impedance matching at higher frequencies. To address this issue, in Step 3, parasitic patch 2 is symmetrically loaded on both sides of the radiating patch to introduce an additional resonance that restores the high-frequency matching. As $l_{j2}$ increases from 1.3 to 1.7 mm, the high-frequency reflection coefficient improves from approximately −10 dB to −17.5 dB, with only a minor upward shift of about 1.5 GHz in the notch band. The final optimized value of $l_{j2}$ balances the tradeoff between high-frequency matching and overall bandwidth. After optimization, the antenna element achieves an operating bandwidth from 18.6 to 45.44 GHz with a notch band from 31.87 to 39.19 GHz.

**Figure 8:**
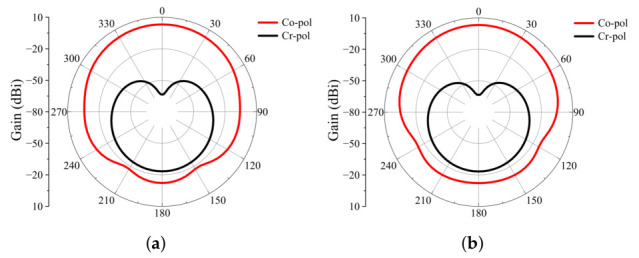
Simulated radiation patterns of the antenna element at 25 GHz: (**a**) E-plane and (**b**) H-plane.

Figure 8 presents the simulated radiation patterns of the antenna element under periodic boundary conditions at 25 GHz. The co-polarized realized gain is 3.21 dBi, and the cross-polarization levels in the boresight direction are better than −45 dB, confirming stable radiation characteristics and excellent polarization purity.

#### 2.1.2. Equivalent Circuit Model

**Figure 9:**
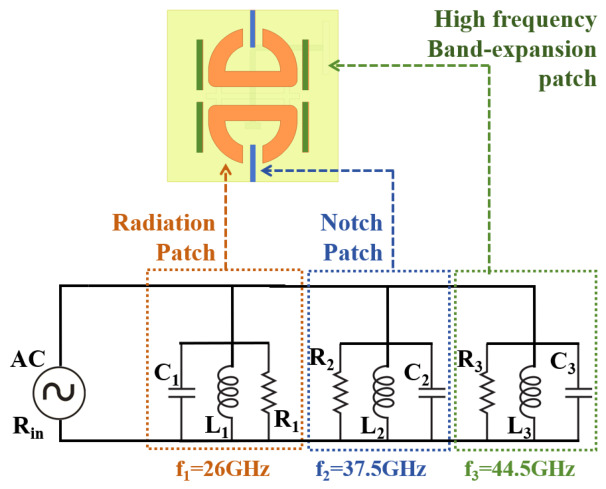
Equivalent circuit model of the proposed antenna element.

To further elucidate the operating mechanism of the proposed element, a simplified equivalent circuit model is developed, as shown in Figure 9. This simplified circuit model is developed to reproduce the dominant resonant features of the proposed element and to provide a physical interpretation of the roles of different radiating and parasitic patches, rather than to replace the full-wave model or to provide an exact point-by-point fitting of all distributed electromagnetic effects. Each functional block of the element is represented by a parallel RLC resonator, and the corresponding component values are listed in Table 2.

The radiation patch, comprising the slot-coupled planar dipole, is represented by the first parallel RLC resonator ($R_{1}$, $L_{1}$, $C_{1}$) with a resonant frequency of $f_{1}=26$ GHz. The H-shaped slot provides a capacitive effect that compensates for the intrinsic inductance of the dipole, generating additional low-frequency resonances and thereby significantly broadening the impedance bandwidth, consistent with the wideband matching observed in Step 1.

The notch patch (parasitic patch 1), placed between the dipole arms, is represented by the second parallel RLC resonator ($R_{2}$, $L_{2}$, $C_{2}$) with a resonant frequency of $f_{2}=37.5$ GHz. At the notch frequency, this resonator presents a near-short-circuit impedance that diverts electromagnetic energy away from the radiating dipole and creates the desired stopband. The very low resistance ($R_{2}=1.5\;\Omega$) reflects the strong resonant trapping effect of the notch branch. This behavior is consistent with the frequency-tunable notch response demonstrated in Step 2 and the surface-current concentration observed on parasitic patch 1 at 38 GHz (Figure 7).

The high-frequency band-expansion patch (parasitic patch 2), loaded on both sides of the dipole, is modeled as the third parallel RLC resonator ($R_{3}$, $L_{3}$, $C_{3}$) with a resonant frequency of $f_{3}=44.5$ GHz. This resonator introduces an additional resonance at higher frequencies, flattening the input impedance across the upper portion of the operating band and thereby recovering the broadband matching that is partially degraded by the notch structure, as confirmed by the high-frequency matching improvement observed in Step 3.

**Figure 10:**
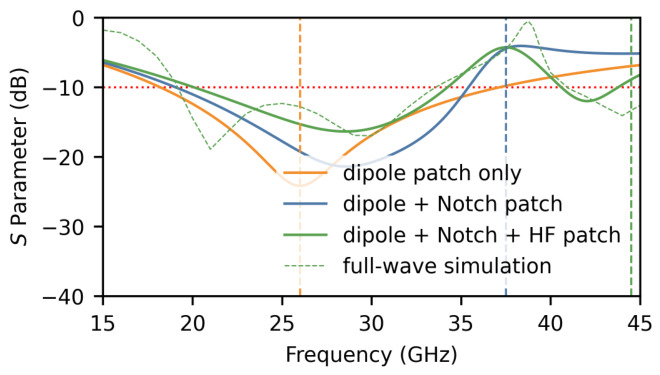
Circuit-simulated responses of the simplified equivalent model with different functional branches. The complete equivalent-circuit response is further compared with the full-wave simulated response of the antenna element.

As shown in Figure 10, the circuit-simulated responses are presented for different functional branches of the simplified equivalent model, and the complete equivalent-circuit response is further compared with the full-wave simulated response of the antenna element. The radiation-patch branch produces the broadband resonance around 26 GHz. After the notch-patch branch is introduced, a stopband response appears around 37.5 GHz. With the further inclusion of the high-frequency expansion branch, the matching is recovered toward the upper operating band above 40 GHz. These stepwise circuit responses are consistent with the design evolution observed in the full-wave simulations in Figure 5.

The complete equivalent-circuit response, represented by the solid green curve, is compared with the full-wave simulated response of the antenna element, represented by the dashed green curve. Although the simplified lumped model does not exactly reproduce every detailed variation of the full-wave result, it shows reasonable consistency in the main resonance locations and the overall notch/matching trend. The remaining differences are mainly attributed to the distributed multilayer aperture-coupled structure, fringing fields, electromagnetic coupling among the metallic patches, the coupling slot and the ground plane, and the finite approximation introduced by the lumped RLC representation. Therefore, the equivalent circuit is used to support the physical interpretation of the dominant resonant mechanisms and functional contributions of different patches, while the final antenna performance is evaluated using full-wave simulation and measurement.

### 2.2. Design of the Feeding Network

**Figure 11:**
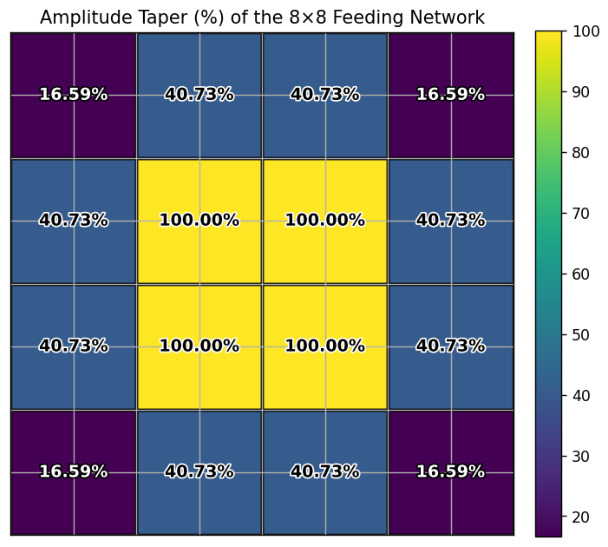
Element excitation amplitudes derived from the Taylor distribution for the array.

To achieve low-sidelobe performance, the feeding network is designed based on a Taylor distribution [25]. The Taylor synthesis is computed using 2×2 subarrays as the basic excitation unit. The calculated excitation amplitudes for one quarter of the 8×8 array are listed in Figure 11; the remaining elements are obtained by symmetry.

**Figure 12:**
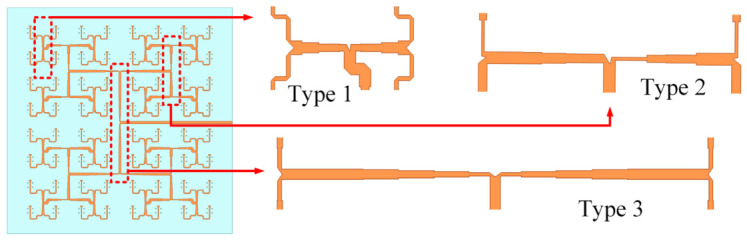
Configuration of the feeding network and the three types of 1-to-4 power dividers.

The feeding-network architecture is illustrated in Figure 12, employing a cascaded configuration of three types of 1-to-4 power dividers to realize the required amplitude distribution over an ultra-wide bandwidth. The Type 1 power divider is a 1-to-4 equal-power divider whose transmission lines are routed with bends to avoid coupling with the H-shaped coupling slot. The Type 2 power divider is a 1-to-4 unequal-power divider, which is the core component responsible for realizing the low-sidelobe amplitude taper; based on the excitation distribution in Figure 11, this divider must achieve a output amplitud ratio of no less than 1:2.45:2.45:4.9 across the operating band. Multi-section impedance transformers are employed in this divider to achieve wideband matching and the required power distribution. The Type 3 power divider is a 1-to-4 equal-power divider that feeds the central subarrays.

**Figure 13:**
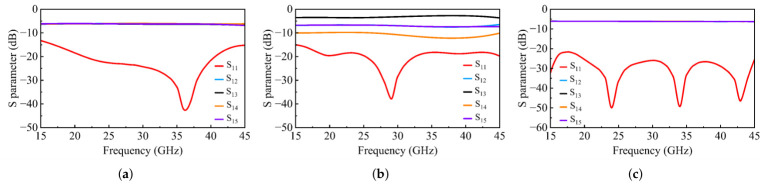
Simulated S-parameters of the (**a**) Type 1, (**b**) Type 2, and (**c**) Type 3 power dividers.

The simulated S-parameters of the three types of power dividers are shown in Figure 13. The optimized Type 1 divider achieves $|S_{11}|\;<-15$ dB from 17 to 45 GHz with good amplitude balance among the four output ports. The Type 2 divider exhibits $|S_{11}|\;<-17$ dB across the target band, with output amplitude ratios closely matching the design specifications. The Type 3 divider achieves $|S_{11}|\;<-20$ dB from 15 to 45 GHz, corresponding to a relative bandwidth greater than 100%, with amplitude imbalance less than ±0.05 dB among the output ports.

**Figure 14:**
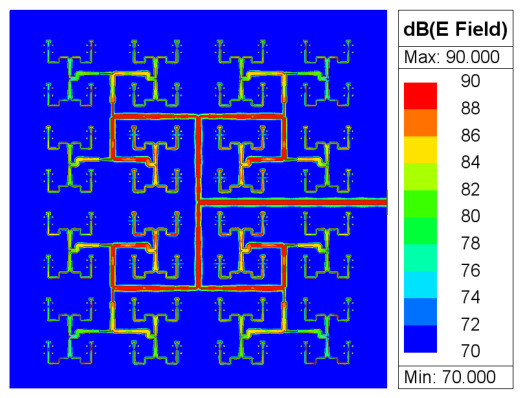
Simulated E-field distribution of the feeding network, showing the amplitude taper consistent with the Taylor synthesis.

Figure 14 shows the simulated E-field distribution of the feeding network. The energy distribution clearly follows the target amplitude taper derived from the Taylor synthesis (cf. Figure 11), confirming the effectiveness of the feeding-network design.

## 3. Results and Discussion

**Figure 15:**
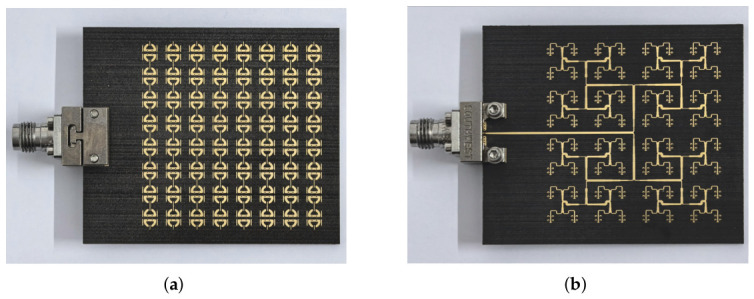
(**a**) Top view and (**b**) bottom view of the fabricated 8×8 array antenna with the assembled RF connector.

Based on the proposed antenna element and the Taylor-weighted feeding network, an 8×8 single-band-notched UWB low-sidelobe planar array antenna has been designed and fabricated. Photographs of the fabricated prototype are shown in Figure 15, and the overall footprint is $45\times 55\;{\mathrm{mm}}^{2}$. The fabricated boards were also inspected by the manufacturer in terms of key dimensional and stack-up parameters, including microstrip line width, line spacing, etched aperture size, board thickness, dielectric-layer thickness, plated-through-hole quality, and copper-plating thickness. In addition to the delivered prototype, an extra fabricated board or process coupon was used for cross-sectional inspection of the multilayer stack-up and via metallization.

**Figure 16:**
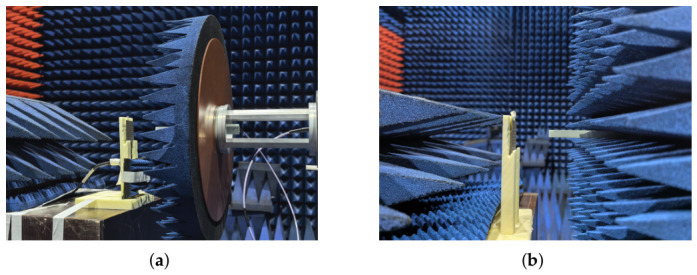
(**a**) Perspective view and (**b**) side view of the measurement setup for the fabricated prototype.

The fabricated prototype was measured in a planar near-field anechoic chamber, as shown in Figure 16. Due to the capability limits of the available measurement facilities, the input reflection coefficient was measured up to 45 GHz, while the near-field scanning system for radiation-pattern and realized-gain characterization was only available up to 40 GHz. Therefore, the measured radiation patterns and gain results are presented up to 40 GHz.

**Figure 17:**
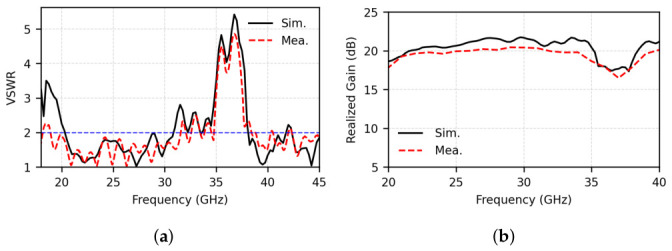
Simulated and measured (**a**) VSWR and (**b**) realized gain of the proposed 8×8 array antenna. The blue dashed line denotes the VSWR = 2 criterion.

Figure 17 presents the simulated and measured VSWR and realized gain of the fabricated 8×8 array antenna. Measured results show that the array covers an overall UWB frequency span from 19.0 to 45.0 GHz, with VSWR<2 in the passbands except for slight ripples, and exhibits a rejection band from 35.0 to 38.5 GHz. These ripples are mainly attributed to the transition-band impedance perturbation before the notch band, where parasitic patch 1 starts to be weakly excited through gap coupling and introduces additional reactive loading to the main dipole. After array integration, this effect is further influenced by mutual coupling, the Taylor-weighted feeding network, and small fabrication or assembly tolerances.

Compared with the element-level response in Figure 5, the notch behavior in the array becomes weaker. The VSWR in the rejected band increases moderately, and the realized gain around 37.0 GHz is reduced by about 5 dB relative to the adjacent passband level. This is expected because the finite 8×8 array, inter-element coupling, edge effects, and feed-network loading modify the effective resonance condition and reduce the Q-factor of the parasitic notch resonator. Nevertheless, the measured VSWR and gain still exhibit a clear depression around 37 GHz, confirming that the proposed parasitic-patch mechanism remains effective after array integration, although with a reduced notch depth in the practical array environment. The measured realized gain is also lower than the simulated value in the higher-frequency band. This discrepancy is mainly attributed to the accumulated loss and tolerance sensitivity of the compact multilayer mmWave array, including conductor and dielectric losses in the cascaded Taylor-weighted feeding network, amplitude/phase imbalance along unequal feed paths, connector and soldering transition loss, material and fabrication tolerances. Therefore, the high-frequency gain discrepancy is considered reasonable for a compact multilayer mmWave PCB array.

**Figure 18:**
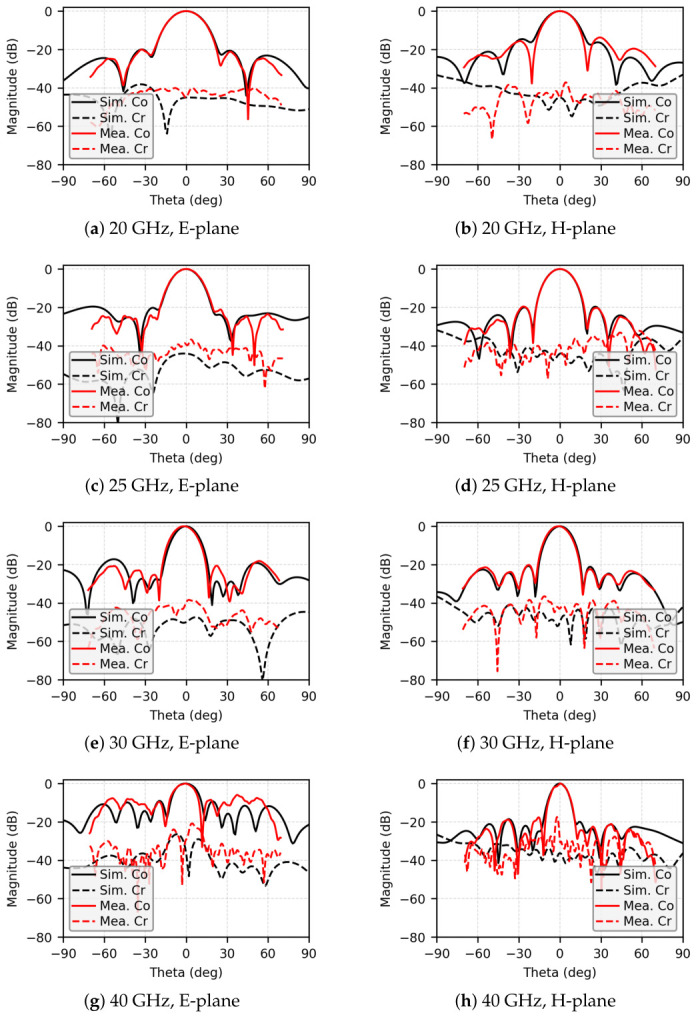
Simulated and measured radiation patterns of the 8×8 array antenna at (**a**,**b**) 20 GHz, (**c**,**d**) 25 GHz, (**e**,**f**) 30 GHz, and (**g**,**h**) 40 GHz.

Figure 18 shows the simulated and measured radiation patterns in both E-plane and H-plane cuts at 20, 25, 30, and 40 GHz. The array exhibits well-formed broadside beams with low sidelobe levels over the passband frequencies. Within the frequency range of 19.0 to 35 GHz, the average sidelobe level in both principal planes remains better than −17 dB. The cross-polarization levels in the boresight direction are maintained below −30 dB over the measured frequencies, indicating excellent polarization purity.

Table 3 compares the proposed array with recently reported UWB and low-sidelobe array antennas. To provide a fairer comparison, the array size or element number is also included, since the peak gain and sidelobe level are closely related to the radiating aperture and the number of elements. It can be seen that some reported works employ larger planar arrays, while others are based on linear or smaller arrays. Therefore, the peak gain should not be interpreted independently of the array size. Compared with the existing designs, the proposed antenna is distinctive because it experimentally combines ultra-wideband operation, a clearly defined 35.0–38.5 GHz rejection band, and low-sidelobe radiation within a single compact 8×8 mmWave planar array. This joint integration of spectral and spatial filtering constitutes the main contribution of the present work.

## 4. Conclusions

A single-band-notched UWB low-sidelobe planar array antenna for mmWave applications has been presented. The element integrates three functional blocks: a slot-coupled planar dipole for UWB impedance matching, a half-wavelength parasitic resonator for controllable notch generation, and side-loaded parasitic patches for high-frequency bandwidth recovery. An 8×8 array with Taylor-weighted feeding achieves an operating bandwidth from 19.0 to 45.0 GHz, a notch band from 35.0 to 38.5 GHz with around 5 dB gain suppression, a peak gain of 20.5 dBi, and average sidelobe levels better than −17 dB from 19.0 to 35.0 GHz. The design demonstrates the feasibility of jointly integrating ultra-wide bandwidth, band-notch functionality, and low-sidelobe radiation in a compact planar array, offering a practical antenna-level pre-filtering solution for interference-resilient mmWave systems requiring suppression around the 37 GHz coexistence region.

## Figures and Tables

**Table 1:** Dimensions of the proposed antenna element (mm).

**Parameter**	*p*	$r_{1}$	$l_{j1}$	$l_{j2}$	$l_{s1}$	$l_{s2}$
**Value**	5.12	0.10	1.10	1.50	2.80	1.00
**Parameter**	$w_{p1}$	$w_{p2}$	$w_{j1}$	$w_{s}$	$w_{f1}$	$w_{f2}$
**Value**	0.46	0.50	0.14	0.16	0.16	0.46

**Table 2:** Component values of the equivalent circuit model.

Branch	*R* (Ω)	*L* (pH)	*C* (pF)	$f_{0}$ (GHz)
Radiation patch ($R_{1}$, $L_{1}$, $C_{1}$)	60	330.6	0.1134	26
Notch patch ($R_{2}$, $L_{2}$, $C_{2}$)	1.5	159.2	0.1132	37.5
HF expansion patch ($R_{3}$, $L_{3}$, $C_{3}$)	50	1073	0.01192	44.5

**Table 3:** Performance comparison with recently reported UWB and low-sidelobe array antennas.

Ref.	Array Size	Freq. (GHz)	Bandwidth (%)	Peak Gain (dBi)	SLL (dB)	Notch
[26]	10-element linear	24–28	15.4	16.1	<−20	No
[27]	16×16	24–30	22.2	18.4	<−17	No
[29]	1×8	24–30	22.2	15.7	<−15	No
[33]	8×8	25–30	18.2	22.5	<−20	No
[42]	1×4	2.5–12.2	131.3	9.4	—	Yes
This work	8×8	19.0–45.0	81.3	20.5	<−17	Yes

## Data Availability

Data are contained within the article.

## Abbreviations

The following abbreviations are used in this manuscript:

UWB	Ultra-wideband
mmWave	Millimeter-wave
SLL	Sidelobe level
VSWR	Voltage standing-wave ratio
SICL	Substrate-integrated coaxial line
SIW	Substrate-integrated waveguide
HFSS	High Frequency Structure Simulator
RLC	Resistor-inductor-capacitor
RF	Radio frequency
BW	Bandwidth
